# Synergetic Effect of Mo, Mg-Modified Sn-β Over Moderate-Temperature Conversion of Hexose to Alkyl Lactate

**DOI:** 10.3389/fchem.2022.944552

**Published:** 2022-07-14

**Authors:** Yanru Hu, Gengrui Zhang, Lele Liu, ZiXin Chi, Shuai Wang, Jingdong Lin, Haifeng Xiong, Shaolong Wan

**Affiliations:** College of Chemistry and Chemical Engineering, Xiamen University, Xiamen, China

**Keywords:** alkaline-earth metal, alkyl lactate, glucose, modified Sn-β, molybdenum, retro-aldol condensation

## Abstract

The thermocatalytic conversion of hexose into valuable chemicals such as methyl lactate under mild conditions is very appealing. Here, we report that Mo, Mg co-modified Sn-β catalyst can effectively catalyze the transformation of glucose and fructose into alkyl lactate at moderate temperatures. A maximum yield of around 35% of methyl lactate was achieved from the conversion of glucose in methanol at 100°C over Sn-β catalyst modified with 3 wt% Mo and 0.5 wt% Mg. However, up to 82.8% yield of ethyl lactate was obtained in the case of fructose in ethanol upon the same catalytic condition, suggesting a significant solvent effect. The Mo species plays a key role to enable the retro-aldol condensation of fructose, in which the competing side reactions are significantly suppressed with the assistance of neighboring Mg species probably through a synergetic effect of Lewis acid-base.

## Graphical Abstract

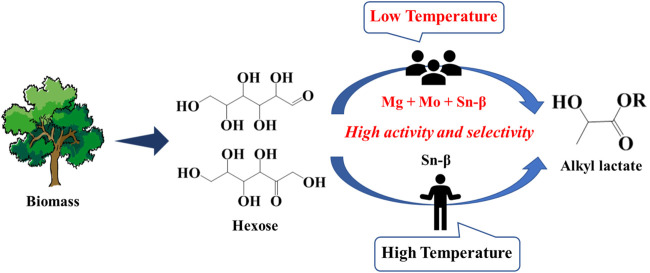

## 1 Introduction

As an abundant, renewable, and nonedible organic carbon resource, lignocellulose biomass is expected to be a promising alternative to fossil resources (Corma et al., 2007; Ong et al., 2019). The carbohydrates, rich in this so-called “second generation” biomass, motivate researchers to devise efficient systems to massively convert them into valuable chemicals, as an appealing way of valorization (Chheda et al., 2007; Hu et al., 2012; Santhanaraj et al., 2014; Gérardy et al., 2020). Among the chemicals, lactic acid/alkyl lactate has been receiving extensive attention, since it can also serve as the feedstock for biodegradable polyester production (Dusselier and Sels, 2014; Mäki-Arvela et al., 2014), other than the applications in food, pharmaceuticals, and cosmetic industries (Datta and Henry, 2006; Eş et al., 2018). As the main approach to current lactic acid production, the fermentation of glucose suffers from some shortcomings, such as low overall productivity, and a complex operation processes (Wasewar et al., 2004; Dong et al., 2016). Alternatively, considerable efforts have been devoted to the development of thermocatalytic processes for lactic acid/alkyl lactate production from carbohydrates over heterogeneous catalysts (Choudhary et al., 2015; Yang et al., 2016; Pang et al., 2017; Wattanapaphawong et al., 2017; Duan et al., 2022).

Amongst the reported heterogeneous catalyst, Sn-containing zeolite is a class of representative solid Lewis acid catalysts (Holm et al., 2010; de Clippel et al., 2012; Holm et al., 2012; Tang et al., 2020). β zeolite with Sn incorporation into the framework as strong Lewis acid sites has been demonstrated to possess good catalytic performance in glucose isomerization to fructose and retro-aldol condensation of C6 carbohydrates at high reaction temperatures (Holm et al., 2010). These two reactions are the key steps involved in the transformation of glucose into alkyl lactate. It is the isolated Sn species in the framework with unsaturated tetracoordinated coordination that exhibit excellent Lewis acidity responsible for the conversion (Li et al., 2011). In addition, our previous work found that the ion exchange with alkaline earth cations (Ca^2+^, Mg^2+^) appears to improve the retro-aldol condensation capacity of Sn-β. It was speculated that the Ca^2+^ or Mg^2+^ neutralized the Brønsted acid sites, thereby mitigating the formation of byproducts. In the meantime, the Ca^2+^ or Mg^2+^ can stabilize the deprotonated alkoxide formed after the proton of the -OH group attached to the C3 of fructose is abstracted by the basic O^2-^ site of Si-O-M (Ca^2+^ or Mg^2+^) (Hu et al., 2020).

Notably, the aforementioned conversions proceeded at high temperatures (>160°C), as the retro-aldol condensation of glucose is insufficient over Sn-β catalysts at moderate temperature. Higher reaction temperatures have an adverse effect on the catalyst durability and need higher requirements for production equipment. Hence, the development of active catalysts is highly desired, in order to achieve a good yield of lactic acid/alkyl lactate at moderate temperatures. In this respect, the pioneering work conducted by Orazov and Davis, 2015. revealed that the hexoses can be converted to ethyl lactate over a mixed catalyst consisting of MoO_3_ and Sn-doping zeolite at around 100°C, achieving a yield of around 70% thanks to the catalytic ability of MoO_3_ toward the retro-aldol reactions. Other oxides like WO_3_ as the co-catalyst mixed with Sn-β were also screened in fructose conversion to methyl lactate, only resulting in an 18% yield at 120°C after 5 h of reaction time (Yang et al., 2019). Supplementary Table S1 presents the catalytic activity of Sn-β and Mo-β-Mg in the present work with the reported literature.

Enlightened by the aforementioned research, herein, we report the Mo, Mg-modified Sn-β catalyst for moderate-temperature conversion of hexose to alkyl lactate. The Mo species loaded on Sn-β zeolite enabled the conversion to proceed at 100°C, thanks to its strong activity for retro-aldol reaction. Moreover, the addition of MgO can tune the Brønsted acid sites and facilitate the rate-determining retro-aldol reaction, the synergetic effects of which with Mo and Sn sites enable the side-reactions limited to a very low extent and thus achieve a superior yield of alkyl lactate.

## 2 Materials and Methods

### 2.1 Materials

Glucose, fructose, mannose, and 5-hydroxymethyl furfural were obtained from Aladdin Reagent Co., Ltd., (Shanghai, China). Tin (II) acetate (99%), methyl lactate (99%), Pyruvic aldehyde dimethyl acetal, magnesium nitrate (99.0%), commercial H-β zeolite (Si/Al = 12.5), HNO_3_ (65%–68%) were supplied by Sinopharm Chemical Reagent Co., Ltd. (Shanghai, China). N_2_ (99.999%) was purchased from Linde Industrial Gases.

### 2.2 Catalyst Preparation

#### 2.2.1 Synthesis of Sn-β

The Sn-β zeolite was prepared by a post-treatment method reported by Hammond et al. (Hammond et al., 2012; Hammond et al., 2015). Specifically, the H-β was treated in concentrated HNO_3_ (65%–68%, 20 mLg^−1^ zeolite) at 333 K for 20 h to remove Al, followed by filtration, being washed thoroughly and dried at 120°C overnight. The resulting solid was denoted as DeAl-β. The solid-state ion exchange method was employed to incorporate Sn into DeAl-β. An appropriate amount of tin (II) acetate was mixed with DeAl-β powder and the mixture was ground manually, and subsequently calcinated at 550°C (10°C/min) in a N_2_ flow for 6 h and airflow for another 3 h. The obtained sample was denoted as Sn-β.

#### 2.2.2 Synthesis of Mo-Modified Sn-β, Mg-Modified Sn-β and Mo, Mg-Modified Sn-β

Both Mo-modified Sn-β and Mo, Mg-modified Sn-β were prepared by the incipient wetness impregnation method. Briefly, 1 g of Sn-β was added with ammonium molybdate solution under vigorous stirring. Then, the mixture was placed for 6 h, followed by drying overnight at 120°C and calcinated at 550°C for 5 h. The resulted solid was referred to Mo modified Sn-β and denoted as Mo-Sn-β. The same procedure mentioned earlier was employed to prepare the Mg-modified Sn-β catalyst, except replacing the ammonium molybdate with magnesium nitrate. With the Mo-Sn-β as the support, the same procedure above was employed to prepare the Mo, Mg-modified Sn-β catalyst, except replacing the ammonium molybdate with magnesium nitrate.

#### 2.2.3 Synthesis of Mo-β and Mg-Modified Mo-β

Both the Mo-β and Mg-modified Mo-β were prepared by the incipient wetness impregnation method. Briefly, 1 g of DeAl-β was added with ammonium molybdate solution under vigorous stirring. Then, the mixture was placed for 6 h, followed by drying overnight at 120°C and calcinated at 550°C in airflow for 6 h. The resulted solid was referred to as Mo-β and denoted as x% Mo-β. With the Mo-β as the support, the same procedure as before was employed to prepare the Mg-modified Mo-β catalyst, except replacing the ammonium molybdate with magnesium nitrate.

### 2.3 Catalyst Characterization

X-ray powder diffraction (XRD) patterns were recorded on Rigaku UItima IV X-ray diffractometer with Cu Kα radiation source (40 kV and 30 mA) from 5 o to 60 o and a scan speed of 10^o^/min. The adsorption-desorption isotherms of N_2_ were obtained on a Micromeritics ASAP 2020 M instrument. The surface area was calculated via the Brunauer–Emmett–Teller (BET) equation, and the volume of pores was acquired *via* the single point method. X-ray photoelectron spectra (XPS) were recorded with a Quantum 2,000 Scanning ESCA Microprob instrument (Physical Electronics) using Al Kα radiation. The binding energy was calibrated by using the C1s photoelectron peak at 150 eV as a reference. Quantification of Mo and Mg elements was conducted using a Bruker S8 Tiger X-ray fluorescence (XRF) spectrometer. Fourier transform infrared (FTIR) spectra were collected on a Nicolet 6,700 instrument equipped with an MCT detector at a spectral resolution of 4 cm^−1^. A self-supporting pellet made of the sample was placed in the flow cell and evacuated under reduced pressure at 623 K for 1 h. After cooling to 373 K, the samples were saturated with pyridine vapor and then evacuated. Subsequently, spectra were recorded at the same temperature in the 4,000–650 cm^−1^ range by using the coaddition of 64 scans.

### 2.4 Catalytic Evaluation and Product Analysis

The reactions were conducted in a batch-type Teflon-lined stainless steel autoclave reactor with an inner volume of 50 ml. Typically, the glucose and catalyst were added to 20 ml of methanol. Then the autoclave was sealed, purged with N_2,_ and pressurized with N_2_ to 2 MPa. Subsequently, the system was heated up to 373 K. After continuous magnetic stirring (800 rpm) for a given time, the autoclave was cooled to room temperature in cold water, and the liquid was obtained after filtration and analyzed by GC and HPLC. Sample products were first qualitatively analyzed using an Agilent GC-MS equipped with a 1701-ms column. The crude product was dissolved in a silylating agent (bis-(trimethylsilyl) trifluoroacetamide +1% trimethylchlorosilane). This solution was maintained at 65°C for 2 h to ensure the complete silylation which was subsequently injected into the GC-MS. Some peaks in Agilent HPLC were further identified using LC-MS. Quantitative analysis was then performed on an Agilent HPLC system equipped with RI and UV-Vis detectors and a Bio-Rad Aminex HPX-87H ion exclusion column (300 × 7.8 mm), using 0.005 M H_2_SO_4_ as the mobile phase at a flow rate of 0.6 ml min^−1^. The column temperature and the detector were both set at 50°C. The number of varied products was determined using calibration curves generated with standard solutions.

## 3 Results and Discussion

### 3.1 Catalyst Characterization

#### 3.1.1 X-ray Powder Diffraction

**FIGURE 1:**
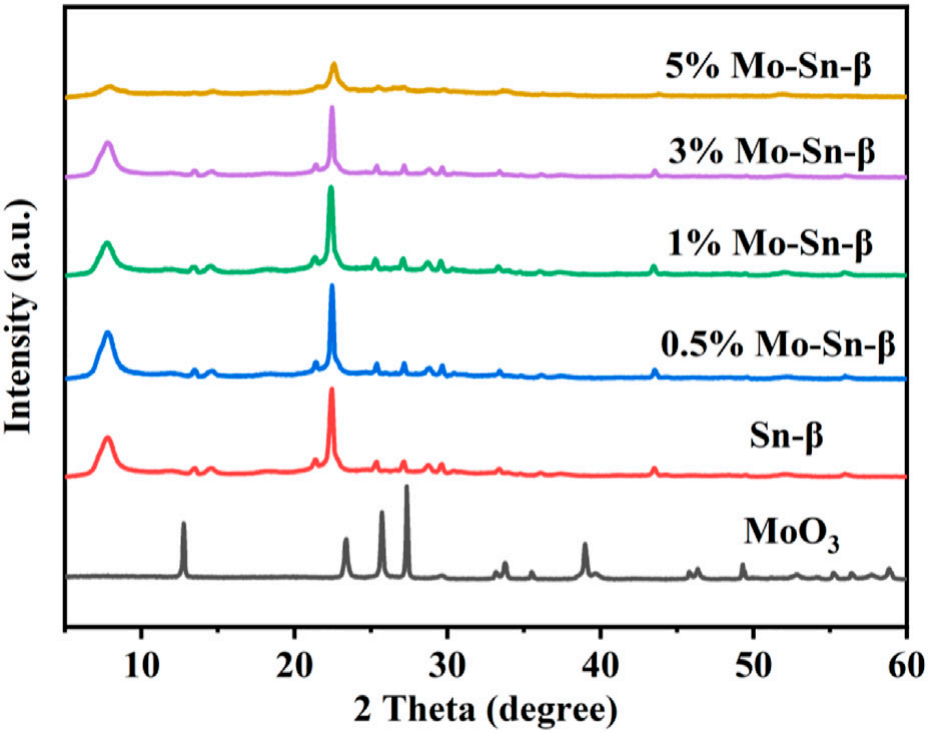
XRD patterns of Mo-modified Sn-β.

The XRD diffraction patterns for the prepared Sn-β modified by different contents of Mo are displayed in Figure 1. The peaks appearing at 27° and 34°, corresponding to (021) and (111) of MoO_3_ respectively, were observed for 5 wt% loading content of Mo. This is probably caused by the aggregation of excessive Mo on the surface of the catalyst. For the samples in which the loading contents of Mo were below 5 wt%, the peaks attributed to the MoO_3_ phase were not observable, indicating the good dispersion of Mo species on the surface.

#### 3.1.2 Brunauer-Emmett-Teller

Brunauer–Emmett–Teller (BET) gas sorptometry measurements were conducted to examine the pore nature of Mo-modified Sn-β catalysts. As listed in Table 1, at Mo contents below 5 wt%, the BET surface area of Mo-modified Sn-β catalysts did not vary significantly, with values in the range of 413–425 m^2^ g^−1^. In contrast, the pore volume decreased by approximately 25% as the Mo content increased from 0 to 5 wt%, which may result from the blocking of pores by the excessive Mo species.

**TABLE 1:** Physicochemical properties of Mo-modified Sn-β.

Catalyst	S_BET_ (m^2^ g^−1^)^a^	V(cm^3^ g^−1^)^a^	Mo content (wt%)^b^
0.5% Mo-Sn-β	423	0.41	0.6
1% Mo-Sn-β	411	0.39	0.9
3% Mo-Sn-β	420	0.35	2.8
5% Mo-Sn-β	417	0.29	5.1

a S_BET_ = Brunauer-Emmet-Teller surface area, V = pore volume.

b Determined by XRF.

#### 3.1.3 X-ray Photoelectron Spectra

**FIGURE 2:**
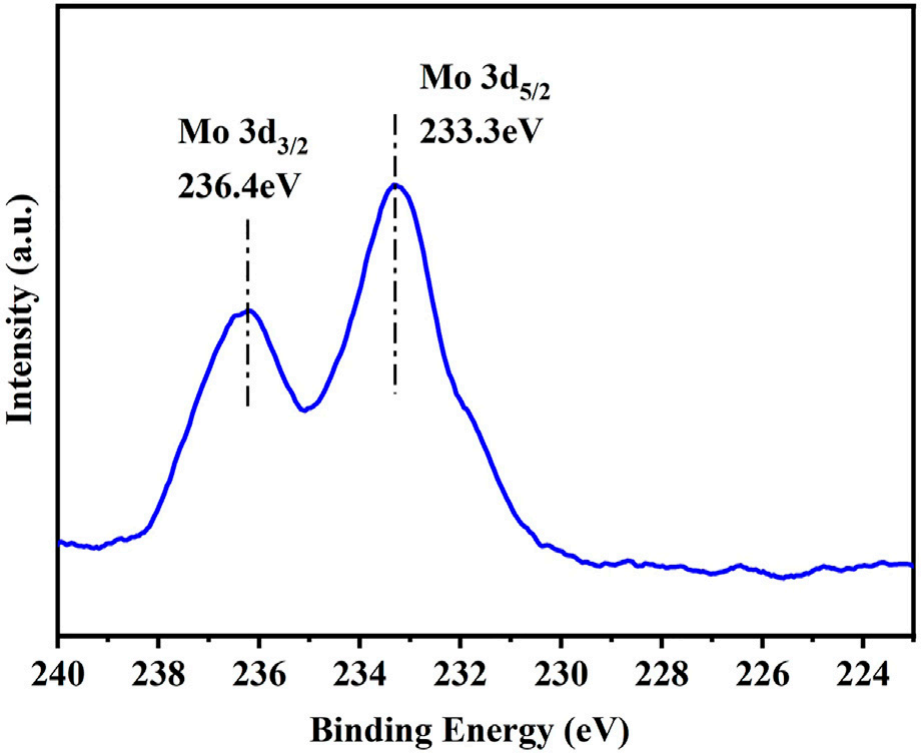
Mo 3 days XPS spectrum of Mo-modified Sn-β catalyst.

X-ray photoelectronspectroscopy (XPS) was used to characterize the Mo oxidation state. The Mo 3 days spectrum exhibits two peaks, located at 236.5 and 233.3 eV respectively, which can be assigned to Mo^6+^ species, as shown in Figure 2. Previous work has verified that Mo^6+^ species possess better catalytic performance in the retro-aldol reaction of fructose than Mo^4+^ (Orazov and Davis, 2015).

### 3.2 Catalytic Performances

**FIGURE 3:**
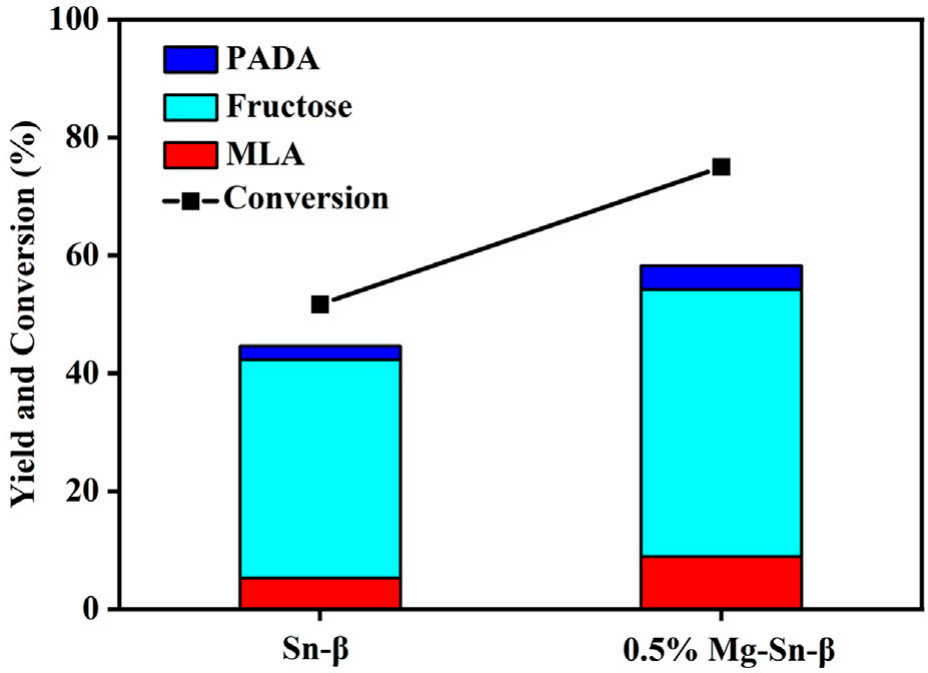
Catalytic performance of Sn-β catalysts and Mg-modified Sn-β catalysts at moderate temperature. Reaction conditions: glucose, 0.055 g; catalyst, 0.4 g; methanol, 20 ml; N_2_ 2 MPa; 4 h; 100°C.

The initial catalytic tests were performed over Sn-β catalysts and Mg-modified counterparts at moderate temperatures. As present in Figure 3, in the absence of Mg modification, fructose was the primary product and accompanied by the production of a small amount of PADA (Pyruvic aldehyde dimethyl acetal), and the yields of methyl lactate were below 10% at 100°C after 4 h over Sn-β catalysts. Comparatively, the Mg-modified Sn-β catalysts promoted the glucose conversion remarkably but still failed to increase the selectivity toward methyl lactate remarkably. The main product was also fructose, implying that the Sn sites alone can catalyze the glucose isomerization to fructose, but exhibited a rather limited catalytic activity for the retro-aldol reaction of fructose at 100°C.

**FIGURE 4:**
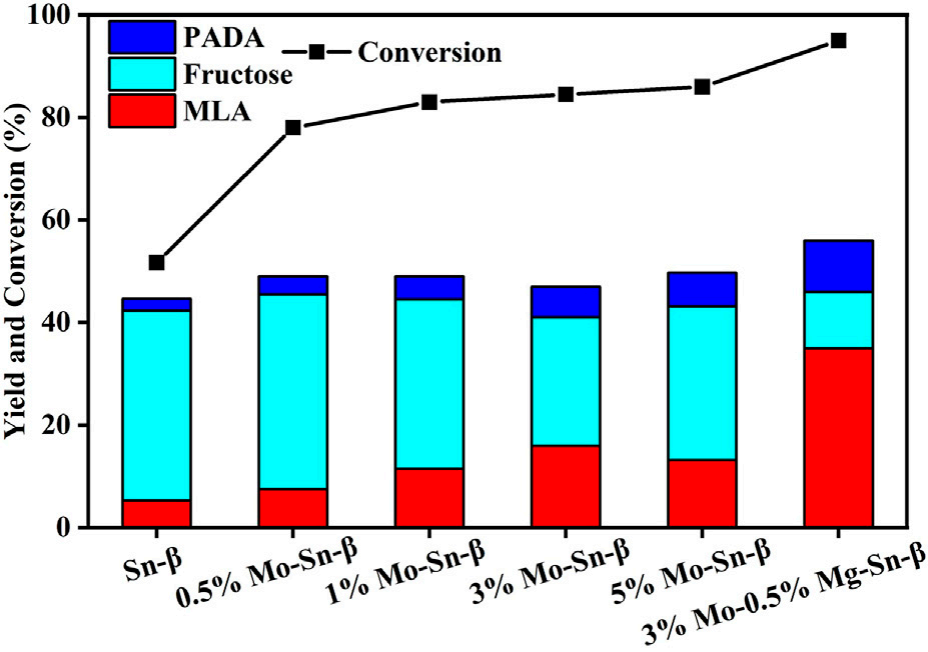
Moderate-temperature catalytic activity of Mo-modified Sn-β catalysts. Reaction conditions: glucose, 0.055 g; catalyst, 0.4 g; methanol, 20 ml; N_2_, 2 MPa; 4 h; 100°C.

The influence of Mo modification on the catalytic performance of the Sn-β catalyst is shown in Figure 4. Without Mo modification, merely a 5% yield of methyl lactate with a glucose conversion of 51.7% was achieved. By contrast, the glucose conversion was increased by above 30% over Sn-β loaded with Mo catalysts, accompanied by promotion in methyl lactate (MLA) yield to a significant extent. The MLA yield first increased and then decreased with the loading content of Mo, reaching a maximum of 16% at 3 wt% of loading content. The 3 wt% Mo enabled the MLA selectivity to increase from 10.3% to 18.9%, revealing that Mo^6+^ species promoted the retro-aldol condensation of fructose to C3 intermediates for the formation of MLA. However, Mo content exceeding 3 wt% resulted in a decrease in both MLA yield and selectivity, which is probably because the excessive Mo species blocked the pores of Sn-β and thus hindered the access of fructose to the Sn sites as reflected in Table 1. Surprisingly, 36.8% of selectivity and 35% yield of MLA were achieved over Mg, Mo co-modified Sn-β catalyst, outperforming the sole Mg or Mo modified counterparts. This implied that a synergistic effect exists between Mg and Mo promoters.

**FIGURE 5:**
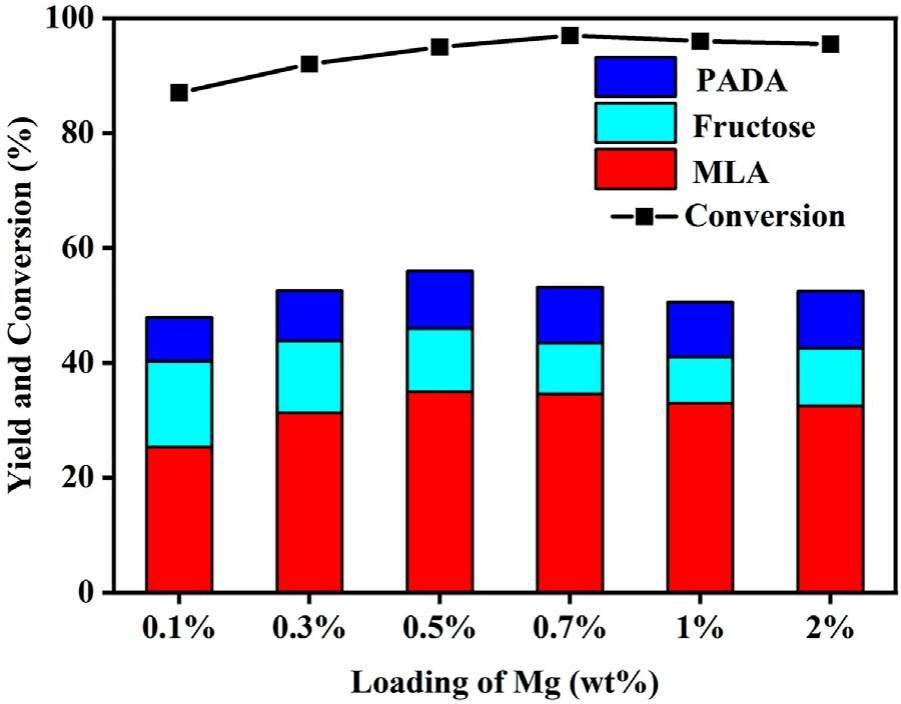
Effect of loading amount of Mg on the moderate-temperature catalytic activity of Mo-modified Sn-β catalysts. Reaction conditions: glucose, 0.055 g; catalyst, 0.4 g; methanol, 20 ml; N_2_, 2 MPa; 4 h; 100°C.

Given that the modification of Mg is capable of facilitating the MLA formation, the effect of Mg content on the MLA yield was further investigated. As present in Figure 5, with Mo content fixed at 3 wt% and Mg content range of 0.1%–2%, the maximum yield of MLA was attained at 0.5 wt% Mg. The higher content of Mg cannot further contribute to the MLA formation.

The catalytic performances at various reaction temperatures were also studied, and the results are shown in Supplementary Figure S1. The MLA yield was increased by elevating the reaction temperature in the range of 100–160°C over all the Sn-β catalysts, regardless of the modification. The highest MLA yield was achieved at 160°C over 3% Mo-0.5% Mg-Sn-β catalyst. This suggests that the kinetic-relevant retro-aldol condensation step is further favored at a higher temperature, against the competing side reactions. However, the structure of zeolite would be undermined after such severe reaction conditions, which limits its practical application (Hu et al., 2020). We did perform the recycling test to evaluate the durability of the catalyst at 100°C. After each run, the spent catalyst was recovered from the reaction mixture, which was subsequently washed with deionized water, dried overnight, and calcinated at 550°C for 5 h in a muffle furnace. Compared to the fresh catalyst, the spent catalyst after four cycles showed a slight loss of activity. This result suggests that the catalyst is relatively stable under moderate temperature conditions (Supplementary Figure S2).

To understand the catalytic function of distinct function sites in-depth, we conducted the conversion of different substrates in the presence of various catalysts within a short reaction time. As depicted in Table 2, upon glucose as the substrate, no MLA formed over the 3% Mo-β catalyst, indicating that the Mo species alone is unable to catalyze the glucose conversion to MLA. A 25% yield of fructose, accompanied by a 2% yield of MLA, was obtained over Sn-β suggests that the Sn species is capable of catalyzing the glucose isomerization but less for retro-aldol condensation of fructose. The latter is the kinetic-relevant step in MLA formation. The co-existence of Sn and Mo species enabled an increase in both glucose conversion and MLA yield, indicating that both species are responsible for glucose isomerization. Regarding the fructose substrate, a higher fructose conversion was observed over 3% Mo-Sn-β catalyst, compared to that over Sn-β catalyst. The PADA also formed during the reaction. These results clearly suggest that Mo species can effectively catalyze the retro-aldol condensation. Nevertheless, without the assistance of Sn sites, the MLA can hardly form. This is because the C3 intermediates (1,3-dihydroxyacetone and pyruvaldehyde) generated through the retro-aldol reaction of fructose still need to undergo a 1,2-H shift reaction prior to MLA formation, which has to be catalyzed by the isolated Sn sites featured of strong Lewis acidic property. Unfortunately, the Mo species does not possess the corresponding catalytic capacity, thus the employment of which alone can hardly produce any MLA. This speculation was further confirmed by the results using 1,3-dihydroxyacetone (DHA) as the substrate. A distinct difference exists in MLA yield and selectivity provided by Sn-β catalyst and 3% Mo-β catalyst, respectively. The former catalyst afforded a selectivity of 68.4% toward MLA, while merely 2.5% was obtained over the latter catalyst. The addition of 3% Mo into Sn-β even caused a notable decrease in MLA yield, further confirming the argument made earlier.

**TABLE 2:** Conversion of different substrates catalyzed by modified or unmodified Sn-β catalysts.

Catalyst	Substrate	Conv. (%)	Yield (%)			
			MLA	Fructose	Mannose	PADA
3% Mo-β	Glucose	40	—	7	13	6
Sn-β	Glucose	36	2	25	—	2
3% Mo-Sn-β	Glucose	73	6	30	12	4
			MLA	Glucose	Mannose	PADA
3% Mo-β	Fructose	29	1	—	—	8
Sn-β	Fructose	16	3	2	—	2
3% Mo-Sn-β	Fructose	33	7	1	—	3
MLA						
3% Mo-β-	DHA	80	2			
Sn-β	DHA	95	65			
3% Mo-Sn-β	DHA	100	47			

Reaction conditions: substrate, 0.055 g; catalyst, 0.4 g; methanol, 20 ml; N_2_, 2 MPa; 1 h; 100°C.

It should be noted that the amount of substrate used in the tests above is quite low, which may cause concern over its potential practical application. Hence, we also performed a series of tests with much larger ratios of substrate/catalyst amount. The results in Supplementary Figure S3 show that comparable MLA yields as earlier were obtained, even with the glucose amount increased to 0.25 g and the catalyst reduced to 0.16 g.

**FIGURE 6:**
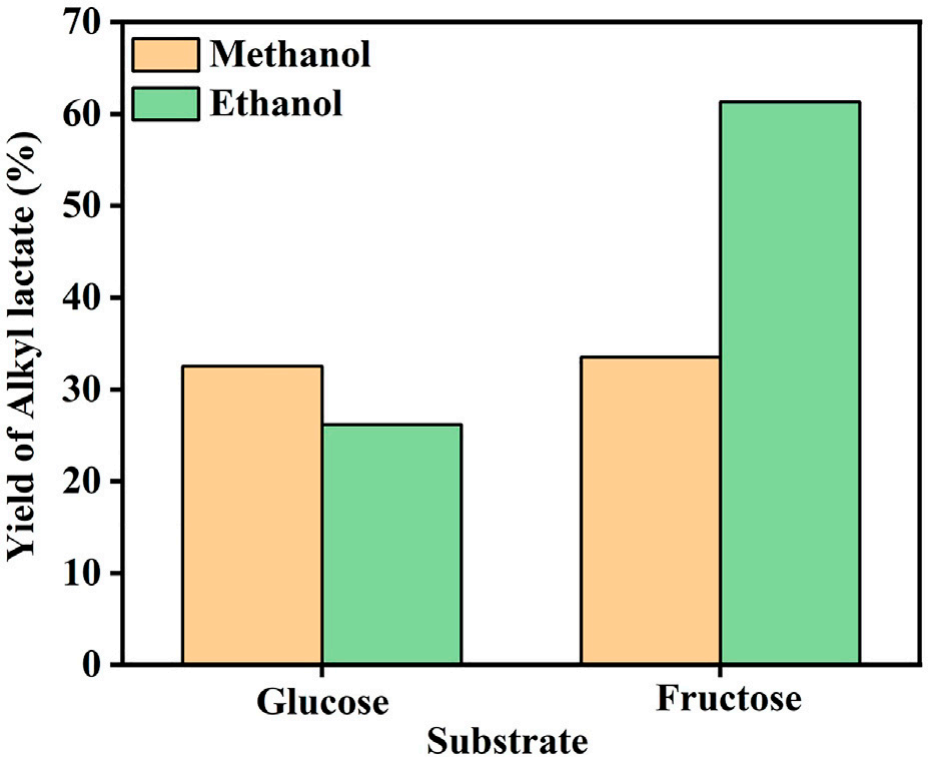
Effect of solvent on the alkyl lactate yield over 3% Mo-0.5% Mg-Sn-β catalyst. Reaction conditions: substrate, 0.15 g; 3% Mo-0.5% Mg-Sn-β, 0.15 g, solvent, 20 ml, 100°C, N_2_, 2 MPa, 20 h.

It has been reported that the use of varied solvents, such as methanol and ethanol, can make quite a notable difference over the yield of the desired products (Orazov and Davis, 2015). In our work, the solvents appeared not to affect the formation of alkyl lactate significantly upon glucose as the substrate but did cause a drastic change in the case of fructose, as exhibited in Figure 6. The yield of alkyl lactate is only about 25% in methanol solvent, while reached up to 61.3% in ethanol solution. Further experiments of fructose conversion over Sn-β and 3% Mo-β catalyst were conducted, respectively. The results in Supplementary Figure S5 show that in the presence of Sn species alone, the fructose conversions in methanol and ethanol were quite similar, while Mo species alone provided a much higher conversion of fructose in ethanol than that in methanol. It was then speculated that the Mo sites were more prone to be affected by the solvent, rather than the Sn sites. Mo species exhibited a lower catalytic performance in retro-aldol condensation of fructose in methanol. It may be attributed to the stronger binding of Mo sites with methanol, hindering the absorption and turnover of fructose on Mo sites.

The aforementioned results demonstrate the indispensable role of Mo and Mg over the modified Sn-β catalysts toward the moderate temperature conversion of C6 monosaccharide. Further experiments show that the physical mixture of 0.5% Mg-β and Sn-β only resulted in about 5% ELA, suggesting neither of them can effectively catalyze the retro-aldol condensation at the moderate temperature. Moreover, the combination of 0.5% Mg-β and 3% Mo-Sn-β could achieve an ELA yield of 54%, which is less than that (61.3%) of 0.3% Mo-0.5% Mg-Sn-β. This demonstrated that the MgO species should stay in close proximity to Mo or Sn sites to have a synergy effect and enhance the yield of ELA. A question then arises whether the Mg species have a synergy effect mainly with Mo or Sn sites in the working catalyst. Hence, we prepared Sn-β, 3% Mo-β and their Mg-modified counterparts to shed light on the intrinsic mechanism. Indeed, the physical mixture of Sn-β and 3% Mo-β in ethanol results in a relatively lower ELA yield at 52%, thanks to the absence of Mg species. The combination of 0.5% Mg-Sn-β and 3% Mo-β can improve the yield of ELA to 60%, comparable to that of 0.3% Mo-0.5% Mg-Sn-β. Thanks to the lack of catalytic ability for the retro-aldol condensation reaction, the addition of basic MgO in Sn-β could probably tune the Brønsted acid sites near the Sn sites, thus mitigating the side-reactions from fructose and intermediate products such as DHA/GLA during the conversion (Hu et al., 2020). However, the employment of Sn-β and 0.5% Mg-3% Mo-β can achieve the highest yield up to 70%, which can be further improved to nearly 82.8% with the extended reaction time (Supplementary Figure S4). This result is superior to most of the prior works reported in the literature, as compared in Supplementary Table S1. It appears that MgO species can cooperate more effectively with Mo-site during the rate-determining retro-aldol reaction step, thus significantly mitigating the side reactions. The weakened Brønsted acid sites near the Mo sites should play a role to abate the production of side products, similar to that of Mg-Sn-β. Pyridine-probed FTIR analysis could readily distinguish the Bronsted and Lewis acid sites. As shown in Supplementary Figure S6, the strength of Bronsted acid was weakened after adding Mg to the 3% Mo-Sn-β catalyst. Our earlier work shows that MgO species attached to the zeolite framework can have a synergy effect with the Lewis Sn site to directly enhance the retro-aldol condensation at a more severe temperature like 160°C. Through functioning as Lewis acid-base pair, MgO can facilitate the abstract of the proton from the OH groups of fructose and stabilize the deprotonated alkoxide that is subsequently subjected to retro-aldol condensation catalyzed by the strong Lewis Sn sites (Hu et al., 2020). It is speculated that a similar mechanism can take place between MgO and the nearby Mo sites in this work, where fructose can be readily deprotonated and stabilized for the subsequent retro-aldol condensation. Hence, the side reactions were further restricted to a very limited extent, leading to a much higher yield of ELA than the other cases listed in Table 3.

**TABLE 3:** Catalytic performance of a physical mixture of modified β catalysts at moderate temperature.

**Entry**	**Catalyst**	**Yield of ELA (%)**
1	0.5% Mg-β + Sn-β	5
2	0.5% Mg-β + 3% Mo-Sn-β	54
3	Sn-β + 3% Mo-β	52
4	0.5% Mg-Sn-β + 3% Mo-β	60
5	Sn-β + 0.5% Mg-3% Mo-β	70

Reaction conditions: substrate, 0.15 g fructose; catalyst, (0.15 g + 0.15 g); ethanol, 20 ml; N_2_, 2 MPa, 100°C; 20 h.

**SCHEME 1:**
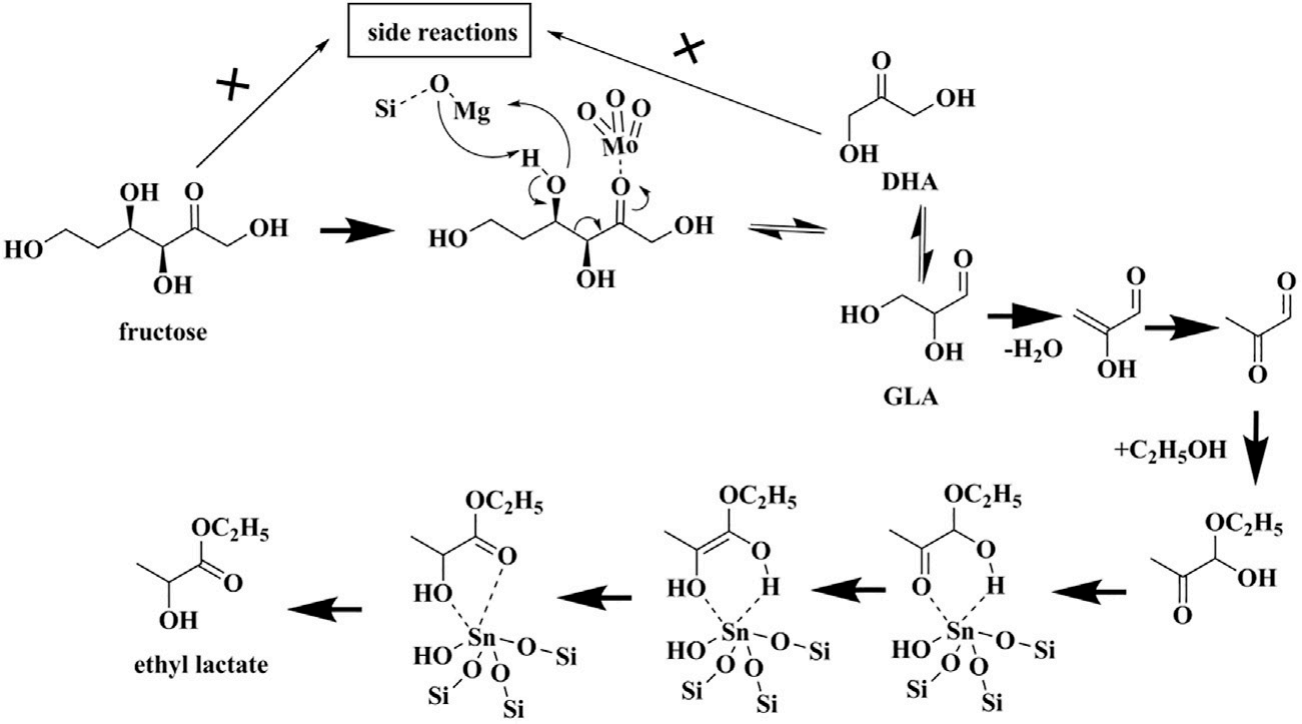
The plausible mechanism of fructose conversion to ethyl lactate over Mo, Mg-co-modified Sn-β catalyst.

Based on the results above, a mechanism is proposed as Scheme 1 below. The ketonic oxygen of fructose adsorbs onto the Mo sites first, followed by the occurrence of retro-aldol condensation to generate 1,3-dihydroxyacetone (DHA) and glyceraldehyde (GLA). In this process, the framework O atom of Si-O-Mg, as the Lewis base site, assists to abstract the H atom of -OH attached to the C3 of fructose, while Mg^2+^ is the Lewis acidic site can stabilize the deprotonated alkoxide. A keto-enol tautomerization exists between DHA and GLA. The formed GLA undergoes dehydration and addition reaction with ethanol molecule, generating the hemiacetal compound 1. Subsequently, compound 1 is transformed into ethyl lactate *via* 1,2-H shift as catalyzed by Sn sites. The side reactions are significantly suppressed during the whole process, thanks to the addition of MgO species.

## 4 Conclusion

The Mo, Mg co-modified Sn-β catalysts are capable of effectively catalyzing the hexose conversion to alkyl lactate at moderate temperature. Especially, an 82.8% yield of ethyl lactate can be achieved from fructose with a combination of 0.5% Mg-3% Mo-β and Sn-β at 100°C. The framework Sn sites are mainly responsible for the isomerization of glucose and 1,2-H shift of formed trioses, while the Mo species mainly account for the C3-C4 bond cleavage of fructose to generate 1,3-dihydroxyacetone and glyceraldehyde (trioses) via retro-aldol condensation. The presence of MgO in close proximity to Mo sites can tune the nearby Brønsted acid sites and facilitate the retro-aldol condensation, thus limiting the side reactions and greatly enhancing the ELA yield.

## Data Availability

The raw data supporting the conclusion of this article will be made available by the authors, without undue reservation.

## References

[B1] Chheda J. N. Huber G. W. Dumesic J. A. (2007). Liquid-Phase Catalytic Processing of Biomass-Derived Oxygenated Hydrocarbons to Fuels and Chemicals. Angew. Chem. Int. Ed. 46 (38), 7164–7183. 10.1002/anie.200604274 17659519

[B2] Choudhary H. Nishimura S. Ebitani K. (2015). Synthesis of High-Value Organic Acids from Sugars Promoted by Hydrothermally Loaded Cu Oxide Species on Magnesia. Appl. Catal. B Environ. 162, 1–10. 10.1016/j.apcatb.2014.05.012

[B3] Corma A. Iborra S. Velty A. (2007). Chemical Routes for the Transformation of Biomass into Chemicals. Chem. Rev. 107 (6), 2411–2502. 10.1021/cr050989d 17535020

[B4] Datta R. Henry M. (2006). Lactic Acid: Recent Advances in Products, Processes and Technologies - a Review. J. Chem. Technol. Biotechnol. 81 (7), 1119–1129. 10.1002/jctb.1486

[B5] de Clippel F. Dusselier M. Van Rompaey R. Vanelderen P. Dijkmans J. Makshina E. (2012). Fast and Selective Sugar Conversion to Alkyl Lactate and Lactic Acid with Bifunctional Carbon-Silica Catalysts. J. Am. Chem. Soc. 134 (24), 10089–10101. 10.1021/ja301678w 22550936

[B6] Dong W. Shen Z. Peng B. Gu M. Zhou X. Xiang B. (2016). Selective Chemical Conversion of Sugars in Aqueous Solutions without Alkali to Lactic Acid over a Zn-Sn-Beta Lewis Acid-Base Catalyst. Sci. Rep. 6 (1), 26713. 10.1038/srep26713 27222322 PMC4879548

[B7] Duan Y. Luo Q. Nie R. Wang J. Zhang Y. Lu T. (2022). Catalytic Conversion of Glycerol to Methyl Lactate over Au-CuO/Sn-Beta: The Roles of Sn-Beta. Catalysts 12 (1), 104. 10.3390/catal12010104

[B8] Dusselier M. Sels B. F. (2014). Selective Catalysis for Cellulose Conversion to Lactic Acid and Other α-Hydroxy Acids. Sel. Catal. Renew. Feed. Chem. 35, 85–125. 10.1007/128_2014_540 24824728

[B9] Eş I. Mousavi Khaneghah A. Barba F. J. Saraiva J. A. Sant'Ana A. S. Hashemi S. M. B. (2018). Recent Advancements in Lactic Acid Production - a Review. Food Res. Int. 107, 763–770. 10.1016/j.foodres.2018.01.001 29580545

[B10] Gérardy R. Debecker D. P. Estager J. Luis P. Monbaliu J.-C. M. (2020). Continuous Flow Upgrading of Selected C2-C6 Platform Chemicals Derived from Biomass. Chem. Rev. 120 (15), 7219–7347. 10.1021/acs.chemrev.9b00846 32667196

[B11] Hammond C. Conrad S. Hermans I. (2012). Simple and Scalable Preparation of Highly Active Lewis Acidic Sn-β. Angew. Chem. Int. Ed. 51 (47), 11736–11739. 10.1002/anie.201206193 23042488

[B12] Hammond C. Padovan D. Al-Nayili A. Wells P. P. Gibson E. K. Dimitratos N. (2015). Identification of Active and Spectator Sn Sites in Sn-β Following Solid-State Stannation, and Consequences for Lewis Acid Catalysis. ChemCatChem 7 (20), 3322–3331. 10.1002/cctc.201500545 26583051 PMC4641460

[B13] Holm M. S. Pagán-Torres Y. J. Saravanamurugan S. Riisager A. Dumesic J. A. Taarning E. (2012). Sn-Beta Catalysed Conversion of Hemicellulosic Sugars. Green Chem. 14 (3), 702–706. 10.1039/C2GC16202D

[B14] Holm M. S. Saravanamurugan S. Taarning E. (2010). Conversion of Sugars to Lactic Acid Derivatives Using Heterogeneous Zeotype Catalysts. Science 328 (5978), 602–605. 10.1126/science.1183990 20431010

[B15] Hu W. Chi Z. Wan Y. Wang S. Lin J. Wan S. (2020). Synergetic Effect of Lewis Acid and Base in Modified Sn-β on the Direct Conversion of Levoglucosan to Lactic Acid. Catal. Sci. Technol. 10 (9), 2986–2993. 10.1039/D0CY00089B

[B16] Hu X. Wu L. Wang Y. Mourant D. Lievens C. Gunawan R. (2012). Mediating Acid-Catalyzed Conversion of Levoglucosan into Platform Chemicals with Various Solvents. Green Chem. 14 (11), 3087–3098. 10.1039/C2GC35961H

[B17] Li P. Liu G. Wu H. Liu Y. Jiang J. G. Wu P. (2011). Postsynthesis and Selective Oxidation Properties of Nanosized Sn-Beta Zeolite. J. Phys. Chem. C 115 (9), 3663–3670. 10.1021/jp1076966

[B18] Mäki-Arvela P. Simakova I. L. Salmi T. Murzin D. Y. (2014). Production of Lactic Acid/Lactates from Biomass and Their Catalytic Transformations to Commodities. Chem. Rev. 114 (3), 1909–1971. 10.1021/cr400203v 24344682

[B19] Ong H. C. Chen W.-H. Farooq A. Gan Y. Y. Lee K. T. Ashokkumar V. (2019). Catalytic Thermochemical Conversion of Biomass for Biofuel Production: A Comprehensive Review. Renew. Sustain. Energy Rev. 113, 109266. 10.1016/j.rser.2019.109266

[B20] Orazov M. Davis M. E. (2015). Tandem Catalysis for the Production of Alkyl Lactates from Ketohexoses at Moderate Temperatures. Proc. Natl. Acad. Sci. U.S.A. 112 (38), 11777–11782. 10.1073/pnas.1516466112 26372958 PMC4586831

[B21] Pang J. Zheng M. Li X. Song L. Sun R. Sebastian J. (2017). Catalytic Conversion of Carbohydrates to Methyl Lactate Using Isolated Tin Sites in SBA-15. ChemistrySelect 2 (1), 309–314. 10.1002/slct.201601752

[B22] Santhanaraj D. Rover M. R. Resasco D. E. Brown R. C. Crossley S. (2014). Gluconic Acid from Biomass Fast Pyrolysis Oils: Specialty Chemicals from the Thermochemical Conversion of Biomass. ChemSusChem 7 (11), 3132–3137. 10.1002/cssc.201402431 25204798

[B23] Tang B. Li S. Song W.-C. Yang E.-C. Zhao X.-J. Guan N. (2020). Fabrication of Hierarchical Sn-Beta Zeolite as Efficient Catalyst for Conversion of Cellulosic Sugar to Methyl Lactate. ACS Sustain. Chem. Eng. 8 (9), 3796–3808. 10.1021/acssuschemeng.9b07061

[B24] Wasewar K. L. Yawalkar A. A. Moulijn J. A. Pangarkar V. G. (2004). Fermentation of Glucose to Lactic Acid Coupled with Reactive Extraction: A Review. Ind. Eng. Chem. Res. 43 (19), 5969–5982. 10.1021/ie049963n

[B25] Wattanapaphawong P. Reubroycharoen P. Yamaguchi A. (2017). Conversion of Cellulose into Lactic Acid Using Zirconium Oxide Catalysts. RSC Adv. 7 (30), 18561–18568. 10.1039/C6RA28568F

[B26] Yang X. Yang L. Fan W. Lin H. (2016). Effect of Redox Properties of LaCoO3 Perovskite Catalyst on Production of Lactic Acid from Cellulosic Biomass. Catal. Today 269, 56–64. 10.1016/j.cattod.2015.12.003

[B27] Yang X. Zhang Y. Zhou L. Gao B. Lu T. Su Y. (2019). Production of Lactic Acid Derivatives from Sugars over Post-synthesized Sn-Beta Zeolite Promoted by WO3. Food Chem. 289, 285–291. 10.1016/j.foodchem.2019.03.039 30955614

