# Supplementary material for: Synergetic Effect of Mo, Mg-Modified Sn-β Over Moderate-Temperature Conversion of Hexose to Alkyl Lactate
Source: Front Chem. 2022 Jul 14;10:944552. doi: 10.3389/fchem.2022.944552 (PMC9329925; doi:10.3389/fchem.2022.944552)
Supplement: Supplementary file 1 [file DataSheet1.docx]

Supplementary Material

# Supplementary Figures


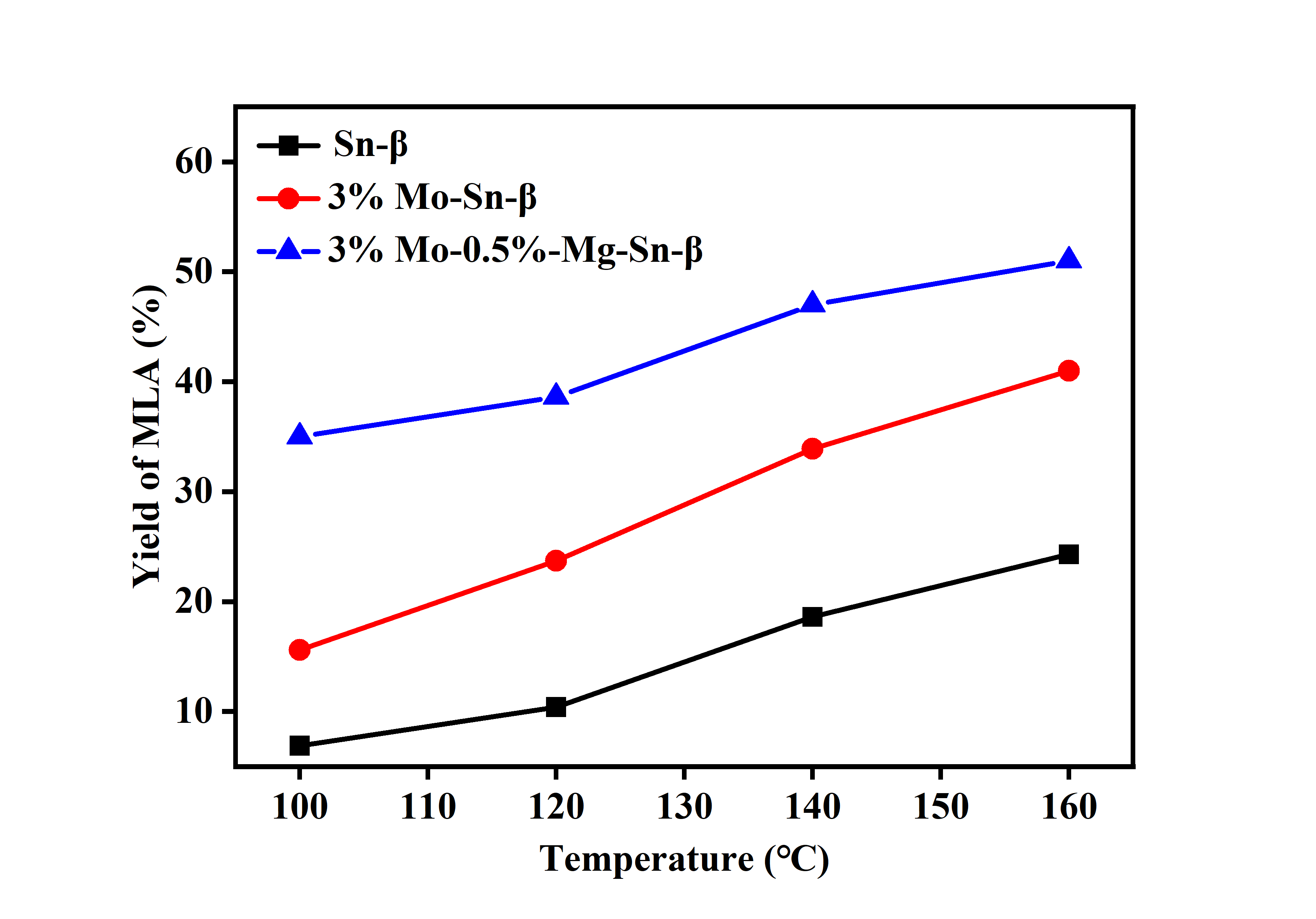


**Supplementary Figure 1.** Effect of reaction temperature on the MLA yield over modified Sn-β catalysts. Reaction conditions: glucose, 0.055 g; catalyst, 0.4 g; methanol, 20 mL; N_2_, 2 MPa; 4 h.


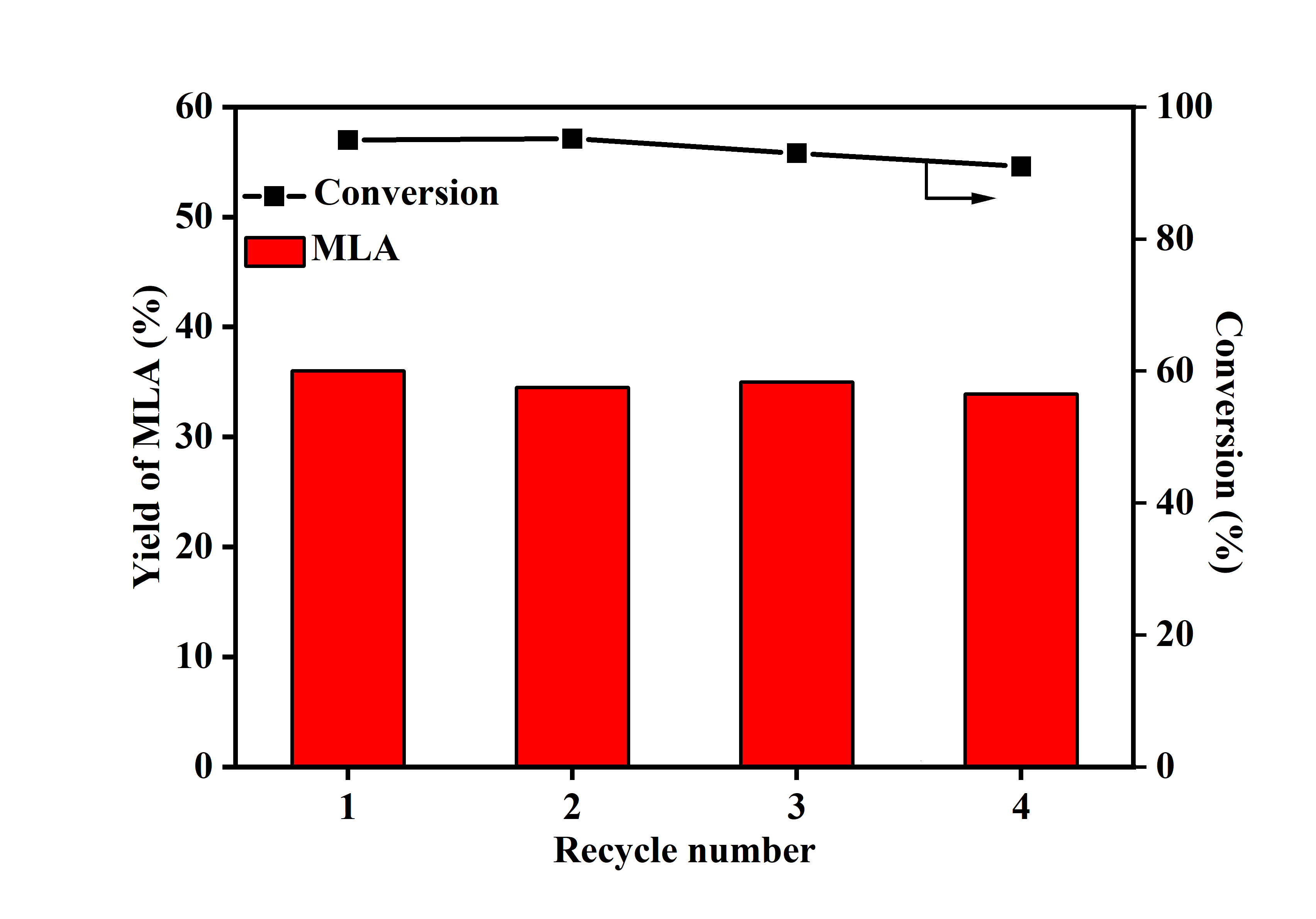


**Supplementary Figure 2.** Recycling test of 3% Mo-0.5% Mg-Sn-β catalyst. Reaction conditions: glucose, 0.055 g; catalyst, 0.4 g; methanol, 20 mL; N_2_, 2 MPa; 1 h; 100 ℃.

**
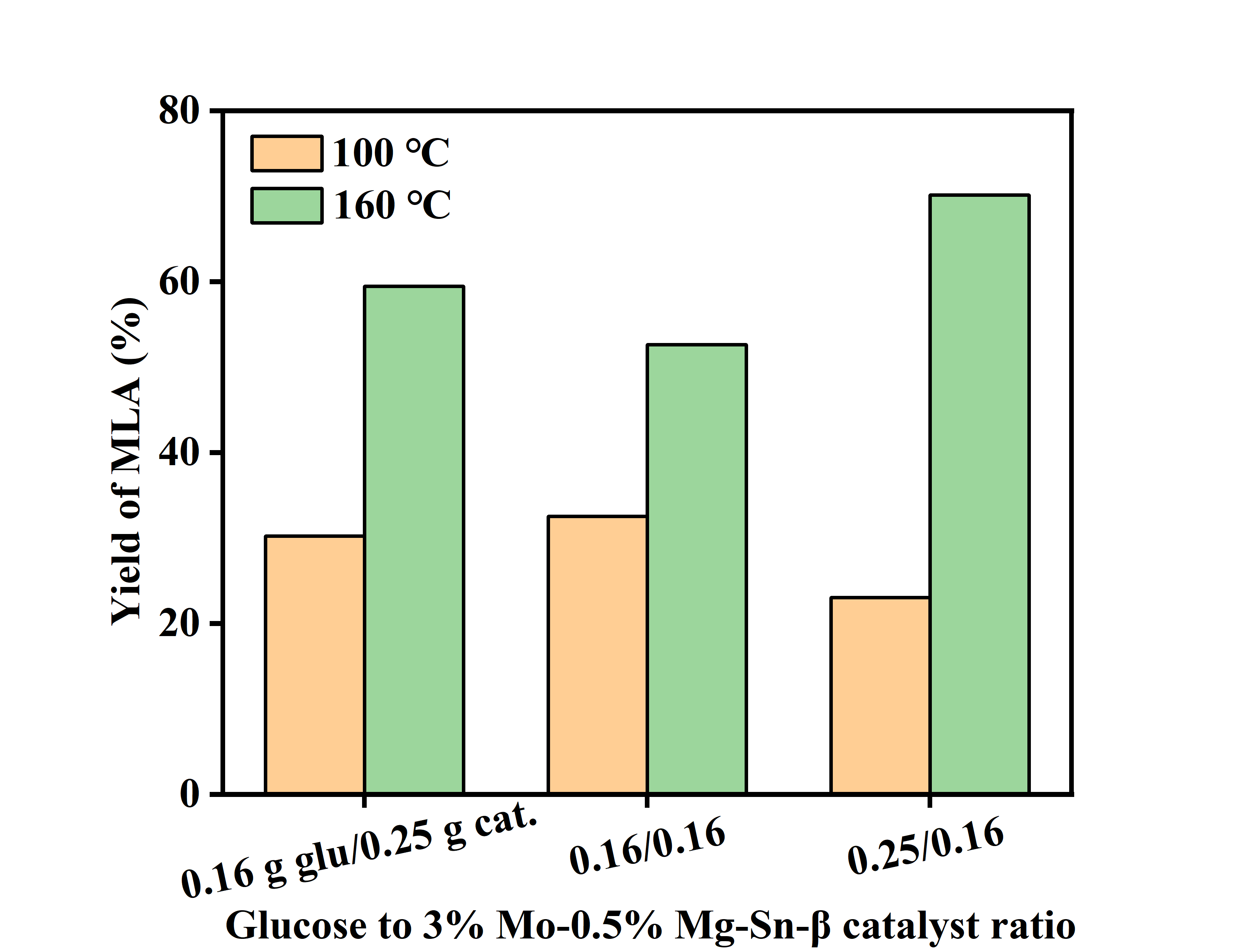
**

**Supplementary Figure 3.** Effect of reaction temperature on the MLA yield of different glucose to 3% Mo-0.5% Mg-Sn-β catalyst ratio. Reaction conditions: methanol, 20 mL; N_2_, 2 MPa; 4 h.

**
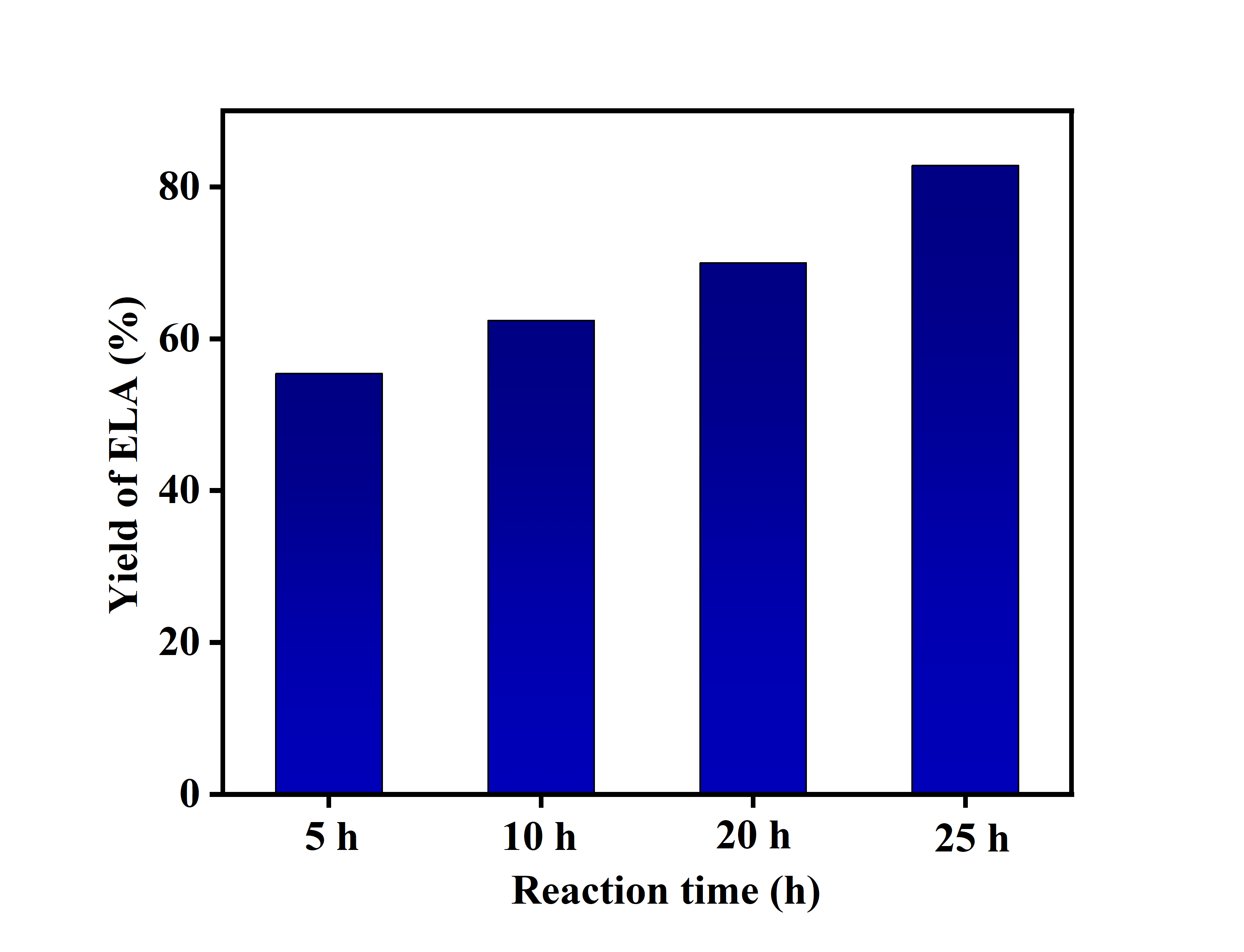
**

**Supplementary Figure 4.** Effect of reaction time on the ELA yield of Sn-β and 0.5% Mg-3% Mo-β catalysts. Reaction conditions: fructose, 0.15 g; catalyst, 0.3 g (respectively 0.15 g); ethanol, 20 mL; N_2_, 2 MPa; 20 h; 100 ℃.


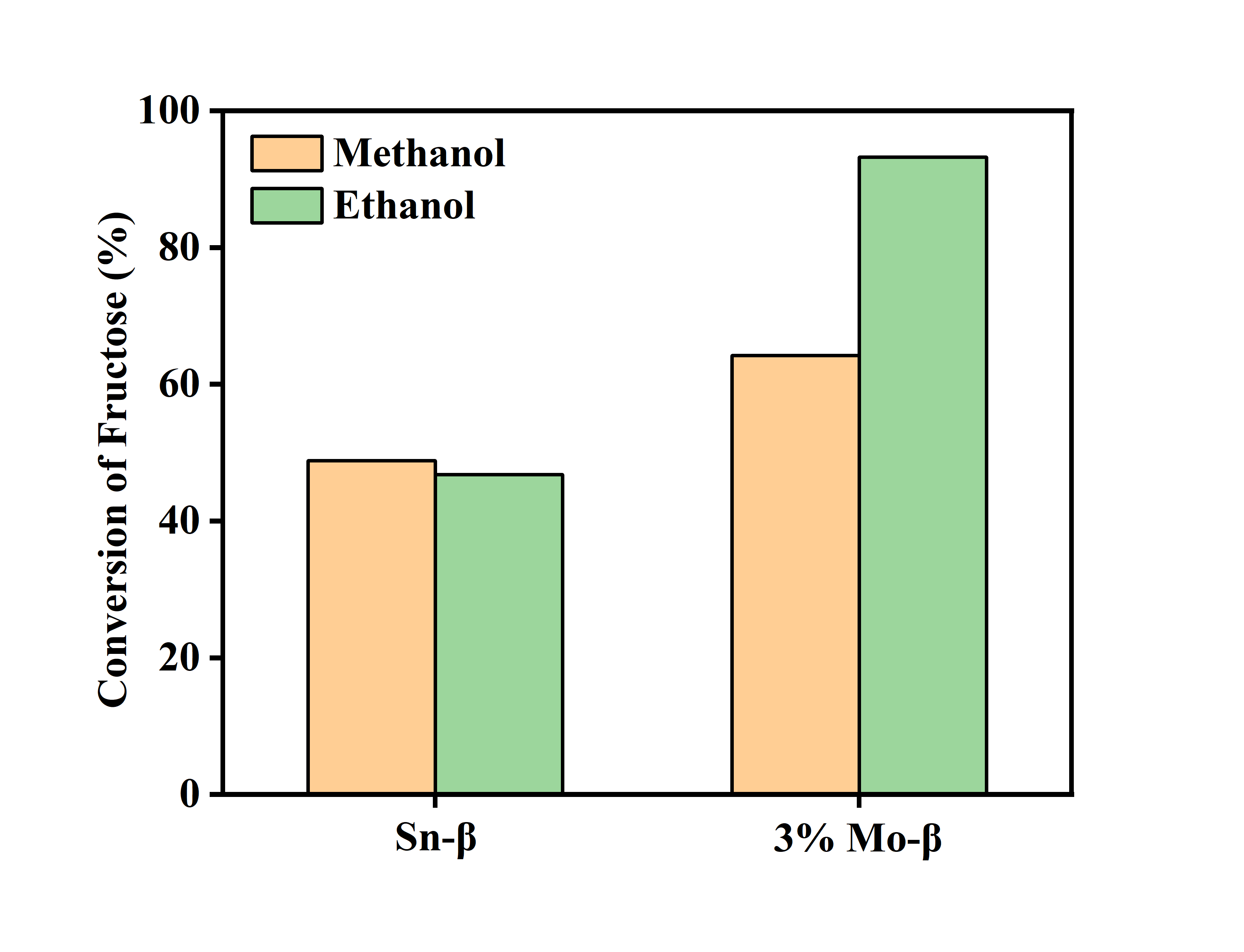


**Supplementary Figure 5.** Effect of solvent on the fructose conversion of Sn-β or 3% Mo-β catalysts. Reaction conditions: fructose, 0.15 g; catalyst, 0.15 g; ethanol, 20 mL; N_2_, 2 MPa; 4 h; 100 ℃.


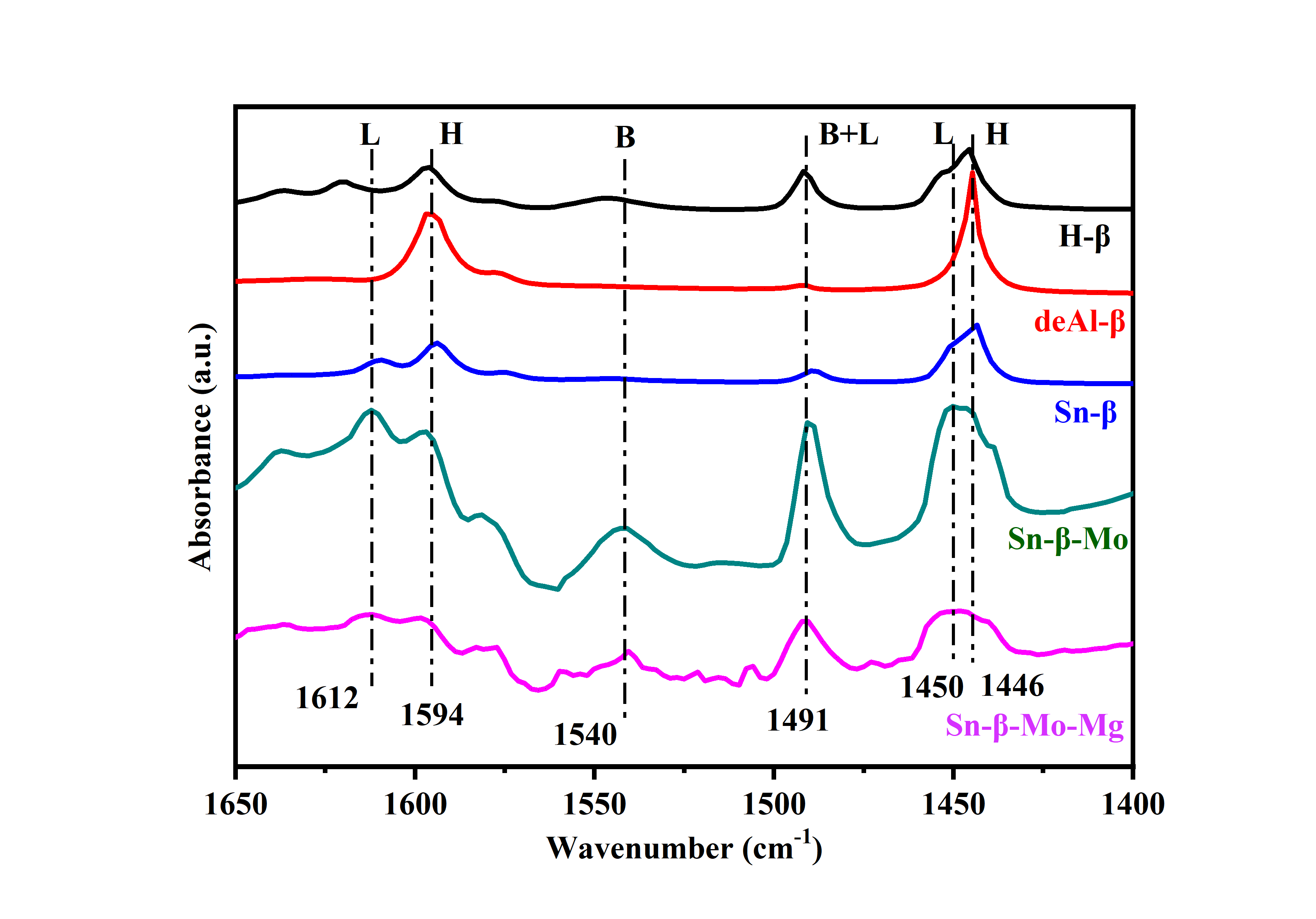


**Supplementary Figure 6.** Pyridine-probed FTIR spectra for Mo, Mg modified Sn-β catalysts. (L: Lewis acid, B: Brønsted acid, H: Si-OH)

# Supplementary Table

**Supplementary Table 1.** Comparative table presenting catalytic activity of Sn-β and Mo-β-Mg in the present work with the reported literature.

| **Entry** | **Catalysts** | **Substrate** | **Temp. (℃)** | **Yield (%)** | **Ref.** |
| --- | --- | --- | --- | --- | --- |
| 1 | Sn-β | Glucose | 160 | 43 (MLA) | Holm et al., 2010 |
| 2 | Sn-SCM-1 | Glucose | 170 | 55 (MLA) | Li et al., 2021 |
| 3 | Zn-Sn-β | Glucose | 190 | 48 (LA) | Dong et al., 2016 |
| 4 | Pd-Sn-β | Glucose | 190 | 52 (LA) | Xia et al., 2018 |
| 5 | Sn-β + SiO_2_ + SiC | Glucose | 180 | 68.3 (MLA) | Liu et al., 2022 |
| 6 | Sn-β + WO_3_ | Fructose | 120 | 18 (MLA) | Yang et al., 2019 |
| 7 | Sn-β + MoO_3_ | Fructose | 100 | 69.2 (ELA) | Orazov et al., 2015 |
| 8 | Sn-β + Mo-β-Mg | Fructose | 100 | 82.8 (ELA) | This work |

# Reference

Holm M S, Saravanamurugan S, Taarning E. (2010). Conversion of Sugars to Lactic Acid Derivatives Using Heterogeneous Zeotype Catalysts. *Science*, 328(5978), 602-605. doi: 10.1126/science.1183990

Li X, Yuan X, Xia G, et al. (2021). Postsynthesis of delaminated MWW-type stannosilicate as a robust catalyst for sugar conversion to methyl lactate. *Industrial & Engineering Chemistry Research*, 60(22): 8027-8034. doi:10.1021/acs.iecr.1c00471

Dong W, Shen Z, Peng B, et al. (2016). Selective Chemical Conversion of Sugars in Aqueous Solutions without Alkali to Lactic Acid Over a Zn-Sn-Beta Lewis Acid-Base Catalyst. *Scientific Reports*. 6, 26713. doi: 10.1038/srep26713

Xia M, Dong W, Gu M, et al. (2018). Synergetic effects of bimetals in modified beta zeolite for lactic acid synthesis from biomassderived carbohydrates. *RSC Advances*, 8, 8965. doi: 10.1039/c7ra12533j

Liu W, Zhou Z, Guo Z, et al. (2022). Microwave-induced controlled-isomerization during glucose conversion into lactic acid over a Sn-beta catalyst. *Sustainable Energy & Fuels*, 6(5): 1264-1268. doi: 10.1039/D1SE01971F

Yang X, Zhang Y, Zhou L, et al. (2019). Production of lactic acid derivatives from sugars over post-synthesized Sn-Beta zeolite promoted by WO_3_. *Food Chemistry*, 289, 285-291. doi: 10.1016/j.foodchem.2019.03.039

Orazov M, Davis M E. (2015). Tandem catalysis for the production of alkyl lactates from ketohexoses at moderate temperatures. *Proceedings of the National Academy of Sciences*, 112(38): 11777-11782. doi: 10.1073/pnas.1516466112
